# Building a presence: implementation strategies used to expand palliative care services across six diverse health systems in a longitudinal mixed methods study

**DOI:** 10.1186/s12904-026-02076-2

**Published:** 2026-04-01

**Authors:** Heather Z. Mui, Laura M. Holdsworth, Karl A. Lorenz, Marcy Winget

**Affiliations:** 1 Division of Primary Care and Population Health, Department of Medicine, Stanford University School of Medicine, 3180 Porter Drive, Palo Alto, CA 94304 USA; 2 Veterans Affairs Palo Alto Health Care System, Center for Innovation to Implementation, Palo Alto, CA USA

**Keywords:** Palliative care, Service expansion, Implementation strategies

## Abstract

**Background:**

Despite the need to increase access to palliative care for aging populations, there is limited research on effective strategies for palliative care service implementation. Identifying implementation strategies that support the integration of services in different clinical settings is a necessary step for the efficient deployment of resources to improve outcomes. This study explores the implementation strategies used by six diverse California health systems to expand palliative care services, and the relationship between strategies and implementation outcomes and reach.

**Methods:**

A longitudinal mixed methods study utilizing document review, key informant interviews, and program data to assess implementation and effectiveness was conducted using a convergent parallel design. A deductive content analysis and matrix analysis using the Expert Recommendations for Implementing Change (ERIC) framework to identify and compare strategies across sites was completed, along with a thematic analysis of the relationship between strategies and reach, feasibility, acceptability, adoption, and sustainability.

**Results:**

In total, 33 of the 73 discrete ERIC strategies were identified, spanning all nine strategy domains. Sites used between 11 and 23 implementation strategies for expanding palliative care programs. All six sites utilized financial strategies, evaluative and iterative strategies, supported clinicians, developed stakeholder interrelationships, and trained and educated stakeholders. We identified four themes that supported outcomes: (1) *establishing and sustaining a trained workforce*, particularly creating new clinical teams with physician leadership made service provision feasible and fostered acceptance and adoption; (2) *identifying cases* was important for referrals; (3) *engaging providers* through relationship development and education was crucial for acceptability, adoption and sustainability; and (4) *involving organizational leadership*, specifically active and enduring executive sponsorship, aided feasibility and sustainability.

**Conclusions:**

Implementation strategies for expanding palliative care services focused on building a presence by establishing a trained workforce to provide the service, identifying cases and engaging providers to increase and sustain acceptability and adoption, involving organizational leadership for feasibility and sustainment, and securing financial support to feasibly launch the service. This study compares how different implementation strategies were used in different settings, and the impact. Palliative care programs looking to expand services should focus on these core strategies to maximize efforts, and reach more patients.

**Supplementary Information:**

The online version contains supplementary material available at 10.1186/s12904-026-02076-2.

## Background

Though recognized as a specialty by the World Health Organization in 1990, access to palliative care is still limited in many settings in the US and globally. Palliative care focuses on physical, psychological, social, and spiritual care to improve quality of life for patients with serious illnesses along with their caregivers [1]. An important function of palliative care is eliciting care preferences to foster goal concordant care [1, 2]. Palliative care has been offered in various settings: inpatient, outpatient, and community (i.e., residential settings, such as homes and skilled nursing facilities). Despite calls for increasing access to palliative care, limited research informs strategies to implement such services to reach the maximum number of patients and caregivers, and how those strategies take shape in the real-world across settings. Although there has been some research around implementation strategies to improve the organization of palliative care [3], most research has focused on supporting the knowledge of appropriate palliative care and integration into oncology services (e.g., [4, 5]). For new clinical practices, particularly referral-based services such as palliative care, identifying and using effective strategies can enhance the likelihood of acceptability and adoption, and program sustainability.

Implementation strategies are crucial to improve uptake and sustain the use of new practices in clinical care [6–8]. Identifying which implementation strategies work in different contexts to achieve meaningful outcomes can provide valuable insights for health providers and inform investments by health systems seeking to achieve similar goals. The Expert Recommendations for Implementing Change (ERIC) strategies and domains offer terminology and a schema to identify and categorize strategies used by implementers [9, 10]. Few studies assess implementation strategies across multiple sites; drawing on experiences from diverse practice contexts can strengthen inference and generalizability of implementation strategies.

In this study, we aimed to understand how health systems employ implementation strategies to implement and embed palliative care services. We used the ERIC implementation strategy taxonomy and domains to characterize and compare implementation strategies across six diverse California health systems undertaking efforts to expand palliative care services. We explored the relationship between strategies and context as well as their impact on reach, defined as the number of patients served.

## Methods

### Design

We conducted a longitudinal mixed methods study of the implementation and effectiveness of different palliative care programs. We assessed program reach, implementation strategies, and implementation outcomes using a convergent parallel design [11, 12]. Quantitative (electronic medical record data) and qualitative (interviews and document review) data were simultaneously collected and analyzed. This study was approved by the Stanford Institutional Review Board, IRB#52693.

### Setting and participants

The Stupski Foundation (the Foundation) funded six health systems in the summer of 2019 to support expansion or enhancement of existing palliative care programs over a three-year period. This investment was made as part of their priority to expand serious illness programs and foster comprehensive care that reduces unnecessary suffering and respects patient wishes. All sites had existing palliative care programs, varying in extent of development and years of operation.

The Foundation also funded the evaluation, and helped connect the researchers (i.e., authors) with study participants. The Foundation identified program leads from the proposals and sent an introductory e-mail, then the researchers reached out to and met with initial program team members, all of whom agreed to be participants in the study as key informants. These key informants shared first-hand experience and insights into the implementation of the expanded services at their respective organization in real-time and facilitated data access. Other informants were sometimes invited by program leads to share their perspectives on the program. Participants typically held operational and/or clinical (i.e. physician, nurse) roles, with a few project support, consultant, or spiritual care representatives.

### Qualitative data collection

#### Interviews

Interviews were conducted with key informants on a monthly or bi-monthly basis, with flexibility based on participant availability and bandwidth, to longitudinally capture program progress and experiences of implementing palliative care services. Interviews were focused on exploring implementation strategies and understanding the context of implementation. Early interviews were semi-structured (see Additional File 1). As interviewers and participants established relationships during repeated interviews, interviews became more conversational. These later interviews focused on project updates and interviewers used their deep knowledge of implementation science to probe deeper in areas of interest (i.e., implementation outcomes, strategies, context). Interviews were conducted via Zoom by trained qualitative health services researchers and implementation scientists (HZM and/or LMH). Interviews typically lasted one-hour, were recorded with permission, and transcribed for analysis.

#### Document review

Each site submitted written grant proposals to the Foundation prior to the research study, which formed the basis of the document review. Proposals included three-year plans with milestones, tasks and activities, and target reach goals. Target goals were used to contextualize program reach while milestones and planned activities supported categorization of implementation strategies and outcomes. These documents were initially reviewed prior to initial interviews with key informants, and referenced periodically during subsequent interviews, with a more formal in-depth review during analysis.

### Qualitative data analysis

Documents and interview transcripts were imported into NVivo (Lumivero, 2020 version) for analysis. Interviews were analyzed on an ongoing basis by the interviewers, with interim reports delivered to the Foundation on a triannual basis. Prior to submitting reports, participants were emailed a summary of key findings and insights to confirm that the program progress and process were accurately being represented and understood. After all the interviews were done, a deductive content analysis [13–15] and matrix analysis [16] were completed to identify and compare strategies used by sites. ERIC strategies [9] offer a detailed taxonomy of 73 discrete implementation strategies that can be used to systematically characterize program implementation efforts. However, the sheer number made it challenging to use as a standalone codebook. Waltz and colleagues [10] grouped these strategies into nine domains, which facilitated the organization and conceptual understanding of the strategies. To enhance usability, we combined the strategies organized under the nine domains from Waltz and colleagues with the definitions provided by Powell and colleagues into one table (see Additional file 2), which along with implementation outcomes [17] was used as a codebook. Two researchers (HZM and LMH) familiar with the data coded transcripts, and met to ensure agreement. Coded data were cross-referenced with the interim reports that participants validated, and organized into matrices and examined in two ways. First, by domain to identify the types of strategies used by sites. Then, assessing the similarities or differences in how strategies were operationalized to explain how implementation strategies related to success. Success was defined by patient reach and implementation outcomes, specifically feasibility, acceptability, adoption, and sustainability. The relationship between implementation strategies and success are presented as themes.

### Quantitative data collection and analysis

Aggregate program data were reported from participating sites, as available. A template with variables to capture patient demographics, clinical characteristics, and service utilization that could be compared across sites was created after discussions with sites. Service utilization was used to assess reach, which was the number of patients who received palliative care from the new service(s) in calendar year 2021 and first six months of 2022. These data were tracked by implementation teams and/or extracted from site electronic medical record (EMR) data reports.

## Results

We conducted 65 longitudinal interviews between July 2020 and May 2022 with 34 implementers from six sites. Table 1 describes key characteristics of the six sites and interview subjects. The settings and types of palliative care expansion were diverse; sites included public/ community hospitals, integrated non- or not-for-profit, and academic and non-academic health systems of varying sizes that served racially, ethnically, culturally, and socioeconomically diverse populations around the San Francisco Bay Area. The number of informants per interview ranged from one to four. Key informants changed over the three years of our study at three sites (B, D, E) due to staffing changes. Moreover, all sites except site E invited additional team members periodically to join interviews to share updates on specific aspects of the programs.

**Table 1:** Site characteristics

Site	Setting description	Inception of existing palliative care services (# of patients served)^1^	Type of palliative care service expansion	Patient characteristics served by the expanded palliative care service in 2021^2^	# of interviews	# of informants (and types of roles^3^)
A	Public/ community hospital: Non-profit community hospital part of small integrated system	Inpatient 2014 (128 patients served)	New: outpatient program	No data; service was suspended and too few patients served.	8	7 (3 administrators, 1 consultant, 1 palliative care provider, 2 project managers)
B	Public/ community hospital: District hospital	Inpatient 2016 (200 patients served)	New: outpatient program	% Non-White: 61% % Non-English: 19% % Medicare: unknown Top diagnosis: 67% cancer	12	7 (2 administrators, 3 palliative care providers, 1 PC care manager, 1 project manager)
C	Public/ community health system: Public hospital system	Inpatient 2008; Outpatient 2016 (400 patients served)	Spread: inpatient programs to two additional hospitals	% Non-White: 73% % Non-English: 22% % Medicare: 60% Top diagnosis: 16% congestive heart failure	9	4 (1 administrator, 2 palliative care providers, 1 project manager)
D	Not-for-profit, large integrated health system	Inpatient 1999; Outpatient 2011 (3,000 patients served)	Spread: community-based program to new county	% Non-White: 48% % Non-English: 10% % Medicare: 86% Top diagnosis: 64% cancer	12	7 (4 administrators, 1 consultant, 1 palliative care provider, 1 project manager)
E	Not-for-profit, large integrated health system	Inpatient 2005; Outpatient 2008 (1,170 patients served)	Pilots: supplemental community-based services	% Non-White: 55% % Non-English: 7% % Medicare: 77% Top diagnosis: 17% neurodegenerative disease	12	4 (3 administrators, 1 consultant)
F	Academic medical center	Inpatient 1999; Outpatient 2015 (2,376 patients served)	Pilot: Augmented outpatient program, tiered service model	% Non-White: 59% % Non-English: 18% % Medicare: 100% Top diagnosis: 28% cancer	12	5 (2 administrators, 1 PC care manager, 2 project managers)

^1^Years indicate when patients were first seen in those settings; most services began in cancer settings and expanded to other disease groups. Number of patients served are annual approximations for 2018/2019

^2^Non-English percentages represent patients whose preferred language is not English. Medicare percentages include Medicare only and Medicare/Medicaid. Top diagnosis is defined as the primary diagnosis with the highest percent among the patients served

^3^Administrators were executives, or operations directors or managers; Consultants were physicians who advised the expanded palliative care service team; Palliative care (PC) providers included physicians and advance practice practitioners (i.e. nurse practitioners) on the new service team; PC care managers were the expanded palliative care team care navigators or chaplains/spiritual care; Project managers, sometimes known as care coordinators, supported the new palliative care services and project-specific activities

Three types of service expansion were observed among the six sites: new program, spread, and pilots. We define new program as a new service in a new setting, namely starting an outpatient service where an inpatient palliative care consultation already exists. Spread is implementing a palliative care service model being used in the health system to a new location, such as one hospital to another hospital or a community-based program from one county to another county. Pilots are experimental palliative care activities that supplement or augment existing services to improve patient and caregiver experiences and/or workflows.

Our findings show different utilization patterns of implementation strategies, operationalized across different palliative care expansion types and settings, with the relationship between strategies and success presented as themes. We first present the types of strategies, or implementation domains, used by each site in Table 2. Then, we share the number of patients served by each site during the study time frame and their target patient reach goals in Table 3. Lastly, four thematic areas characterizing the implementation of palliative care services were identified by exploring the relationship between implementation strategies and reach, feasibility, acceptability, adoption, and sustainability.

**Table 2:** Domains of ERIC strategies used by sites, ordered from most to least used

Site	Setting; type of service	ERIC strategy domains								
		Utilize financial strategies	Use evaluative and iterative strategies	Support clinicians	Develop stakeholder interrelationships	Train and educate stakeholders	Adapt and tailor to context	Change infrastructure	Engage consumers	Provide interactive assistance
A	Public/community hospital; outpatient expansion	x	x	x	x	x			x	
B	Public/community hospital; outpatient expansion	x	x	x	x	x			x	
C	Public/community health system; inpatient spread	x	x	x	x	x	x	x		x
D	Large integrated health system; community-based spread	x	x	x	x	x	x	x		x
E	Large integrated health system; community-based pilot	x	x	x	x	x	x	x		
F	Academic medical center; outpatient pilot	x	x	x	x	x	x	x	x	

**Table 3:** Program reach by site and year

Site	Setting and palliative care (PC) program descriptions	# of patients served, 2021	# of patients served, 2022 (first 6 months)	Target annual reach
A^1^	Public/community hospital expands PC service to outpatient setting	7	-	100
B^2^	Public/community hospital expands PC service to outpatient setting	31	19	173
C^3^	Public/community health system spreads inpatient PC service to two additional hospitals	233 Hospital 1: 150 Hospital 2: 83	196 Hospital 1: 94 Hospital 2: 102	250 Hospital 1: 150 Hospital 2: 100
D	Large integrated health system spreads community-based PC team to serve patients in the most appropriate setting (home/ residence, clinic, and/or hospital)	198	114	300
E^4^	Large integrated health system pilots four community-based PC programs -- *Pilot 1*: hospice discharges and non-admits cared for by hospice team *Pilot 2*: hospice discharges and non-admits cared for by interdisciplinary PC team *Pilot 3*: high social needs patients supported by local navigators *Pilot 4*: unpaid family caregivers of patients with serious illnesses supported by social worker	287 Pilot 1: 56 Pilot 2: 73 Pilot 3: 35 Pilot 4: 123	304 Pilot 1: 69 Pilot 2: 72 Pilot 3: 28 Pilot 4: 135	645 Pilot 1: 120 Pilot 2: 100 Pilot 3: 75 Pilot 4: 350
F^5^	Academic medical center pilots outpatient tiered service model	145		437

^1^Site A’s outpatient service launched February 2020; it was active for 8 months in 2021 and did not operate in 2022

^2^Site B’s outpatient service launched February 2020, and paused for two months in Summer 2021 with a transition in physician lead

^3^Hospital 1 at Site C launched February 2020. Hospital 2 launched September 2021, active for 3 months in 2021

^4^Site E pilots launched at various times to address different gaps in palliative care services. Pilot 1 began April 2021, active for 9 months in 2021. Pilot 2 launched January 2020, active all of 2021. Pilot 3 started May 2021, supporting patients for 8 months in 2021. Pilot 4 launched March 2021, supporting caregivers for 10 months in 2021

^5^The number of patients served by Site F’s pilot program represents the total number served between 2020 and 2022; the target reach was for the 3-year time period, not an annual goal

### Types of strategies

Table 2 shows the breadth of implementation strategy domains utilized by each site. All six sites applied multiple and varied implementation strategies to support palliative care service expansions. Five strategy domains were used by all six sites: (1) *utilize financial strategies*, (2) *use evaluative and iterative strategies*, (3) *support clinicians*, (4) *develop stakeholder interrelationships*, and (5) *train and educate stakeholders*. All sites *utilized financial strategies* by applying to and ultimately receiving funding from the Foundation. Funding made it possible for the launch and implementation of these palliative care services, from employing new palliative care clinicians or care managers to educating non-palliative care clinicians in primary palliative care and the development and testing of caregiver support programs, to contracting with agencies to provide social service navigation and developing a screening tool to identify patients with palliative care needs. Summaries of what sites did and how strategies were operationalized at each site are available in Additional File 3. Similarly, conditions of the funding application included *using evaluative and iterative strategies*, specifically developing a formal implementation blueprint and identifying barriers and facilitators. Formal implementation blueprints outlined planned timelines and program activities, but did not explicitly state implementation strategies that would be used as they were not based on any specific or pre-determined framework. The specific ERIC strategies identified across the six sites to *support clinicians*, *develop stakeholder interrelationships*, and *train and educate stakeholders* were more wide-ranging.

The remaining four implementation strategy domains had mixed use among the sites, though clear utilization patterns by expansion type emerged. Sites spreading or pilot-testing programs (C-F) utilized a*dapt and tailor to context* as well as *change infrastructure* strategies, specifically change record systems [see theme 2 below] and change credentialing; these strategies were used together with *training and educating stakeholders*. Similarly, programs being spread (C, D) also *provided interactive assistance*, which supported the establishment and sustainment of the expanded palliative care workforce [see theme 1 below]. Strategies to *engage consumers* were used by outpatient programs (A, B, F), which informed and educated patients and the community, but alone had limited impact on patient reach.

### Reach

Table 3 details the target annual patient reach alongside the actual reach of each program in the year 2021 and the first six months of 2022. Annual patient reach goals were presented as projections of service use in site proposal documentation. Services launched at various times, thus not all services were active all 12 months of 2021 (see Table 3 footnotes). None of the sites achieved their target in 2021. At the six-month mark in 2022, site C was trending towards achieving their goal, having reached over half the number of patients they aimed to serve annually. Sites A and B had difficulty reaching their outpatient populations, with site A suspending service indefinitely in October 2021.

Sites reported experiencing unforeseen delays and setbacks due to COVID and subsequent health system response. At smaller public/ community hospitals and health systems (sites A-C), staff from the palliative care expansion team rallied to support surge planning, testing, and/or vaccination efforts to best serve their patient communities, which was prioritized above the not yet well-established palliative programs. The one site that expanded into the inpatient setting (site C) was able to capitalize on supporting surge planning and patient care in the hospital during this period, whereas outpatient and community services did not have the same opportunity, due to in-person contact limitations. Notably, sites C-F had more resources at baseline compared to sites A and B, which allowed them to endure the reassignment of roles and loss of staff or pressure to reallocate some staff for pandemic efforts. In the context of the pandemic, larger health systems with intact teams or specialist presence were more protected from adverse effects and better equipped to continue implementation efforts, resulting in greater patient reach than their smaller counterparts.

### Implementation strategies and success

Strategies across all nine domains were used, with 33 of the 73 ERIC strategies operationalized across the six sites. Sites used different combinations of implementation strategies, ranging between 11 and 23 strategies (see Additional File 3). Although none of the sites reached their target patient goal in 2021, some were closer to successfully reaching their goal in 2022 and being able to sustain their service. In comparing strategies across the six sites, we identified four thematic areas that characterized the implementation of the palliative care services. Themes reflect issues related to the programs themselves, the hospital or health system practice setting, and the regional/ecological setting of implementation. Exemplar quotes for each theme are presented in Table 4.

**Table 4:** Exemplar quotes by theme

Theme	ERIC strategies	Exemplar Quotes	Insights and implications
Establishing and sustaining a palliative care trained workforce	Support clinicians: *Creating new clinical teams* Train and educate stakeholders: *Make trainings dynamic*	“Currently we only have two active participants within this program […] Our nurse practitioner, is still the only care provider within this program. We still haven’t got any progress on getting a new palliative care physician.” – Site A (August 2020, Int 24) “I do think to get early buy-in, often, it does take a good physician leadership to really get a program going successfully […] I think there’s just so much variability because there’s not that standard fellowship training [for non-physicians…] You [would] have to find a strong [advance practice provider] who can really lead a program and have the trust of referring physicians.” – Site D (September 2021, Int 70) “Our goal is to do a lot of shadowing and bedside teaching […] because the goal is to get [the nurse champions] to the point that they’re comfortable identifying and starting and following up on consults in conjunction with the advanced practice nurse that will be hired at the end of year 2. […] They’ll stay in their roles when the advanced practice nurse comes. And that will also expand the reach of the service too.” – Site C (September 2020, Int 26)	Site A had a difficult time finding a palliative care physician, hiring an NP that served 7 out of their target 100 patients, ultimately suspending their service. Palliative care physician role was a key piece of fostering trust and acceptability among referring physicians. All sites trained their new non-physician palliative care workforce. Site C also trained nurse champions and had a dynamic training approach, which was in response to delayed trainings due to the pandemic, but felt to be valuable.
Case identification – EMR algorithms vs. provider referrals	Change infrastructure: C*hange record systems*	“We have an algorithm, it generates lists, and then we have the providers review the lists. And that part has been the hardest part this summer, is using that same method. And so, now we’re looking at the physician lead […] and have them review their list in person with the chief of that department, asking them to do that and then getting the time in the meeting to do that, and seeing if that works.” – Site E (September 2021, Int 73)	The academic and large integrated health systems used algorithms to help identify potential patients appropriate for palliative care, but even with the use of algorithms, still required provider engagement and buy-in.
Engaging providers to grow and sustain programs	Train and educate stakeholders: C*onduct educational meetings*,* conduct ongoing trainings*,* make training dynamic* Develop stakeholder relationships: *Identify and prepare champions* Train and educate stakeholders: *Conduct educational outreach visits*,* conduct ongoing trainings*	“I think that there’s some of the physicians that we work with are happy to get recommendations, learn in that way on a patient by patient basis. And then I think that there’s others, and nurses as well, that benefit from a system wide training. This is why we do it this way. And so that we have that first. And so I think it’ll help to be able to address it in both ways. Right now, we just keep a tally of what are all the things that we wish people were doing differently or that they were resistant to doing when we ask them. And I think that will help us to be able to tailor our learning, our education.” – Site C (January 2022, Int 83) “Our referrals have slacked a little bit again from oncology, and we’re starting another round of focused outreaches around certain diagnoses, specifically non-resectable pancreatic cancer patients to get early palliative referrals for those folks to get them in, that’s a place where palliative care has been proven to prolong life by four to six months. So it seems like a pretty easy sell to oncology. We’re in the works of doing some education and outreach to the providers in that way, and also having our new nurse go and do warm relationships with all of the RN case managers for the oncologists.” – Site D (April 2021, Int 57)	All sites engaged and educated providers about palliative care to increase awareness and referrals, though some referrals were inappropriate patients, prompting some sites to offer ongoing educational outreach with tailored messaging. Different sites focused their outreach efforts with different groups, depending on their new service setting and relationships. Those that tracked outreach efforts and/or identified champions (e.g., oncology RN case managers) tailored their efforts and provided ongoing education, resulting in more consistent and appropriate referrals.
Involving organizational leadership	Develop stakeholder relationships: *Involve executive members*	“Because [executive sponsor] was here and stable, we had an administrator who could field a lot of stuff for us. But if she had left too, I actually think things would’ve […] been really, really hard […] I’ve been on other projects where my champion left, and then there was a whole new cadre and nobody cared about all this incredible work we did and it was debunked. So I think in our case, there was stability enough that we were able to cope with those [nursing and executive leadership] changes. – Site C (August 2021, Int 64)	Active and enduring executive member involvement was important for initial and ongoing advocacy for new palliative care programs, especially during institutional changes.

#### Theme 1: establishing and sustaining a palliative care trained workforce

In order to establish and sustain a trained workforce, sites utilized strategies spanning four domains: *support clinicians*, *develop interrelationships*, *train and educate stakeholders*, and *provide technical assistance*. Specific implementation strategies sites used to establish and sustain a trained palliative care team included *create new clinical teams*,* organize clinician implementation team meetings*, *make trainings dynamic*,* and facilitation.*

*Creating new clinical teams* was an essential strategy for all palliative care programs, since expanding services would not be feasible without a dedicated workforce. All grantees proposed establishing new positions, from program support and coordination to physician/advance practice provider roles. While most sites were ultimately able to fill the positions, programs experienced delays due to hiring difficulties, in part due to pandemic-related hiring freezes and altered priorities. Those unable to fully staff teams experienced setbacks in establishing their programs. The two public community hospitals (sites A & B) starting outpatient services had the most challenging time with staffing, in particular finding and retaining qualified palliative care physician candidates to support their diverse, primarily non-English speaking populations; as a result, they reached 7 and 31 patients (out of their annual target goal of 100 and 173), respectively in 2021 during the 8 and 10 months the services were active.

The palliative care physician role was seen as a key piece of outpatient, inpatient, and community home-based services to foster acceptability among physicians to refer patients to palliative care (i.e., adoption). Site A tried to find a bilingual or language-concordant palliative care physician for their mostly monolingual non-English patient population, but realized this was an exceptional expectation, and pivoted to recruiting palliative care physician candidates willing to work with a diverse population from an already limited pool of palliative care specialists. In response to these physician recruitment difficulties, site A hired a nurse practitioner as their outpatient palliative care lead and *developed an academic partnership* for training; however, the implementation team found that referring physicians reported being less inclined to refer their patients to a nurse practitioner and Site A ultimately suspended their service. For sites C and D, once physician leads were onboarded, teams felt the program was on track to be successful and better sustained; in 2021, site C served 233 patients (out of their target goal of 250) on their inpatient services and site D served 198 community-based patients (out of their target goal of 300).

Most programs also included an element of training for their workforce in communication or palliative care skills to upskill their new non-physician palliative care members and/or existing workforce. All sites that hired non-physician palliative care team members provided training and mentorship, as there is no standardized palliative care training for non-physicians. Two sites (C and D) also trained their existing workforce of non-palliative care clinicians on communication strategies or primary palliative care. However, the impact of the COVID-19 pandemic limited initial access to trainings, such as VitalTalk and End-of-Life Nursing Education Consortium (ELNEC). In response to these changes, some sites postponed trainings while others adapted their approach; site C opted to provide in-house mentorship with in-time patient cases and education to new non-physician palliative care team members and nurse champions as an interim measure, which then continued and was considered to contribute to *making training dynamic* and largely felt to be valuable.

Moreover, programs being spread (sites C & D) had additional support through *facilitation* and/or *centralized technical assistance*. Site C had a dedicated centralized implementation team that supported the services and kept the programs accountable, including a facilitating physician consultant as well as a care coordinator who problem solved alongside the palliative care teams, liaising between the team, unit managers, and executive leaders. Site D involved a physician consultant from an existing community-based service in another county for facilitation in guiding and supporting the new team.

#### Theme 2: identifying cases – EMR algorithms vs. provider referrals

For referral-based services such as palliative care, identifying patients who would benefit from the service is a key feature for sustaining programs and providing needed care to patients and their families. The academic and large integrated health systems (sites D, E, F) *changed infrastructure*, specifically record systems. They used algorithms to identify patients potentially appropriate for their services to help maintain a more consistent flow of patients rather than relying solely on provider referrals. These EMR algorithms were helpful, but not sufficient if used in isolation, as they tended to identify a large number of patients who were not deemed suitable for the services following chart review, or who declined services. While algorithms may hold some promise, a definitive denominator for palliative care services has not been determined. Sites that employed algorithms reported that they plan to continue to iterate on their algorithms.

While algorithms can identify a wide range of patients and may relieve some of the burden of patient identification from referring providers, they do not alleviate the need for engagement and support from referring providers. All grantees conducted some form of outreach and education to referring providers to make them aware of the types of patients appropriate for palliative care, or the patients who were identified by the algorithm to check for agreement that the patient would benefit and be amenable to palliative care to increase acceptability and adoption. While labor intensive, all programs, whether utilizing an algorithm or not, undertook outreach and engagement efforts with referring providers (theme 3).

#### Theme 3: engaging providers to grow and sustain programs

All sites needed to socialize their programs and raise awareness within their organizations to garner support and prove their value to physicians in order to generate referrals needed to grow and sustain programs. The palliative care teams conducted outreach to referring providers in ambulatory, acute, or skilled nursing facility (SNF) settings as appropriate, to increase acceptability and adoption (i.e. referral). Strategies to *develop relationships* and *educate stakeholders* were often used in tandem to foster acceptance and generate referrals.

All grantees described having to educate providers and patients/families about palliative care, differentiating it from hospice, and what types of patients are appropriate for referral. Sites reported receiving referrals for inappropriate patients even after outreach, with some sites then offering additional case-specific one-on-one provider education.

Sites recognized the importance of tailored messaging and that ongoing educational outreach were necessary to remind practitioners to make referrals. Several types of educational outreach were provided, including formal *educational meetings* (e.g., Grand Rounds and department meetings) and informal *educational outreach visits* in the practice settings (e.g., huddles and morning rounds). Sites that tracked who they met with and how many referrals were coming from each were able to provide targeted outreach on an ongoing basis, and noticed more consistent and appropriate referrals.

Outreach and educational efforts were part of every program’s implementation plan, differing in frequency and target audience. Most sites were able to conduct some outreach during the three-year period, even though trainings and outreach plans were delayed by the pandemic, which reduced initial referrals and service provision. Those able and diligent in increasing engagement through tailored and repeated educational meetings and/or visits felt more successful and had better patient reach.

#### Theme 4: involving organizational leadership

While the above themes reflect implementation primarily at the program level, we also heard about the importance of support at the organizational level for embedding palliative care. Sites reported that palliative care was perceived to be of value by organizational leaders. However, despite the perception that there is implicit value in palliative care, there was also recognition of the need to demonstrate the business case for palliative care in order to sustain established programs. There were common metrics that sites said were of organizational importance, such as emergency department visits, hospitalizations, 30-day readmissions, and patient satisfaction. However, it was also acknowledged that palliative care does not align well with those commonly used metrics, and that many aspects of palliative care are not easily measured. Most sites seemed to be satisfied with demonstrating that services are cost saving or cost neutral, but more mature programs described the need to potentially make a stronger case for cost effectiveness in the future for continued or adapted service based on pilots to provide palliative care to meet the needs of patients and caregivers in any given setting (inpatient, outpatient, or community).

Initial and continued involvement of *executive members* was a benefit to programs that had ongoing support. Active and enduring executive sponsorship was particularly important during times of executive and program staff turnover and transitions, when advocacy with new leadership and staff budget considerations was needed. Those with executive sponsors who attended regular meetings (sites C – F) had an easier time executing their programs than those with less-involved executives (sites A & B).

In our final round of interviews, the five sites that continued their programs had plans for ongoing work to sustain services, which often included discussions with executive leaders because of the end of the Foundation funded period and need for the organization to absorb costs. Participants noted how it was easier to justify continuing to fund an existing position, which Foundation funds made possible, than advocating for new personnel for a new service or program. The feasibility and sustainability of these expanded palliative care services rely on the support of organizational leadership along with funding, in conjunction with acceptability and adoption by referring physicians for patient referrals, which is supported by continued tailored outreach and necessitated by a trained workforce to provide the care.

## Discussion

Building a presence of newly expanded palliative care services requires funding, a supportive infrastructure with active executive engagement and dedicated team to be feasible and sustainable, along with awareness and understanding among providers for referrals to promote utilization. Sites that successfully *created new clinical teams* were able to provide more comprehensive palliative care for their patient populations; in particular, physician leadership was necessary for acceptance by referring clinicians. As a referral service that serves different people across many disease trajectories, having more visibility and being top of mind among referring providers supports acceptability and adoption. Training and education are often necessary, but rarely a sufficient strategy on their own [3, 18]. While all sites implemented training and educational strategies, those with more tailored approaches tended to see more referrals. During implementation, participants realized that outreach efforts needed to be ongoing to increase or sustain referrals, and emphasized the need for palliative care to be involved earlier in patients’ disease trajectories, which has been shown to improve quality of life and symptom control and reduce hospitalizations [1, 19, 20]. *Developing stakeholder relationships* and *educating stakeholders* worked synergistically; education helped relationship development and maintenance, and relationships facilitated more education opportunities, together increasing acceptability and adoption. Having a trained workforce, educating and developing relationships to get referrals, and involving organizational leadership, along with funding to support these efforts, enabled sites to reach more patients and caregivers who could benefit from palliative care. In comparing the utilization and operationalization of implementation strategies across six different sites, it is clear that these are key strategies, with specific and intentional planning (e.g., not just educating and engaging stakeholders once, but tailored and ongoing education for referring providers and active and ongoing executive engagement), that support building a presence to increase feasibility, acceptability, adoption, and ultimately reach and sustainment.

Adaptations in implementation often occur in the real-world, and the pandemic is an extreme example of changes and competing priorities that both expanding and existing health care services experienced, influencing outcomes. In this particular instance, patient reach was impacted, as all sites were affected by the COVID-19 pandemic and subsequent health system responses. Implementation of these services, and the operationalization of implementation strategies during the pandemic is likely different than how programs would have progressed if not for COVID-19, though it may not be dissimilar from when other changes or disruptions occur, such as institutional changeover. Most sites reported doing what they had set out to do, as outlined in their proposals, with adjusted timelines and adapted strategies, but the program aims and goals remained the same. Programs experienced delays, but there were both positive and negative impacts of the pandemic on existing and expanding palliative care programs [21]. There were conditions such as hiring freezes that implementers may have felt as particularly impactful, but there would have still been a shortage of qualified palliative care physicians. The outpatient and community-based programs were particularly affected by the pandemic, as restrictions prevented patients from accessing or accepting care, dispersed provider attention, and disrupted planned training and education sessions; these restrictions were experienced across all health systems (e.g., [22–25]). Although outpatient palliative care services can start quickly with a small staff compared to inpatient services [26], our sample shows that consistent clinician specialist presence and executive member advocacy are necessary to withstand major disruptions or competing priorities. It is important to consider external pressures, such as organizational changes, local or state policies, and national or global events that influence real-world applications of implementation strategies in establishing practices and expanding services.

Our thematic findings spanned the nine strategy domains, and revealed that some of the 73 discrete strategies were connected. Similar to other studies, we found the ERIC strategies could be enhanced or clarified [27–29], as not all strategies are clear and distinct, nor fit neatly into the assigned domains. Conceptually, multifaceted strategies drew from different domains but were meant for a singular purpose, as demonstrated by our themes (i.e., establishing and sustaining a palliative care trained workforce included six discrete strategies from four different domains). Differentiating program staff training and stakeholder education would be valuable given that *training and educating stakeholders* and *developing stakeholder interrelationships* are often the most frequently used implementation strategies (e.g., [30–32]). Our study highlights how training for program staff and education for referring providers serve different purposes but both training and education should be ongoing; training supports the delivery team to increase service delivery feasibility, whereas education supports acceptability and adoption of the service. We also found that sites implemented strategies in response to changes, indicating the evolution of implementation over time, and the adaptation of new strategies in the face of adversity. Lastly, the identification of ERIC strategies was an academic exercise completed by the researchers, while on the ground implementation teams spoke about implementation strategies as activities or packages of a program. Nonetheless, describing and mapping implementation strategies and outcomes across different sites and contexts over time is valuable in understanding which strategies have been and could be used to address specific needs and desired outcomes in different contexts.

### Limitations

We had monthly or bi-monthly check-ins with implementation teams that enabled close to real-time updates of programs, but still relied on implementer recall and sharing of what was happening and why. Some sites were more forthcoming with details and their reflections on the status of their program than others; thus, perhaps overlooked or unintended implementation strategies were missed. Although interviews were conducted longitudinally, pinpointing when specific strategies were used during which stage of implementation and how, why, and which strategies evolved overtime was difficult and not a focus during data collection and analysis. Future studies and evaluations that select a framework or reporting guidelines to track and map strategies during a project trajectory would be valuable [33, 34]. This sample is unique in that they all applied and received funding from the Foundation to expand palliative care services. Each site developed a plan and submitted proposals that addressed the aims of the Foundation to increase access, reduce suffering and increase goal concordance. Some strategies were embedded in the application process (i.e., *develop a formal implementation blueprint*,* assess for readiness and identify barriers and facilitators*, and *involve executive members*), with the result being *accessing new funding*; whether these strategies reflect organizational initiative and its impact is unclear beyond the funding needed for program feasibility. However, any new program would likely need an outlined implementation plan as well as executive member involvement in order to secure funding and be able to move forward. In our sample, the executive sponsorship required of grantees was a letter of support; theme 4 highlights the importance of more active and ongoing executive engagement over lighter-touch executive involvement. Finally, insights related to program effectiveness were limited due to the difficulty of retrieving broadly comparable clinical and utilization data across these sites with varied program types. The use of each site’s proposed target reach was the best gauge for assessment, along with the broad mapping of implementation outcomes to implementation strategies.

## Conclusions

Palliative care programs looking to expand their services should focus on a few core implementation strategies to maximize their efforts for increasing feasibility, acceptability, adoption, and sustainability to reach more patients. Our findings suggest that *creating new clinical teams* with physician leadership is an essential implementation strategy for fostering trust that supports acceptability and adoption of palliative care programs. Continuous *stakeholder relationship development* and retention, particularly with respect to referring clinicians and leadership, in conjunction with *educational outreach* endeavors, are indispensable strategies to increase reach and support sustainment. Strategies promoting within- and cross-team relationships, education, and support are important for increasing feasibility, acceptability, and adoption and should be ongoing for sustainment. Additionally, securing financial support increases feasibility and is a necessary strategy to expanding services. Future studies and palliative care programs would benefit from intentional selection of implementation strategies and assessment of their impact on implementation and program outcomes. The identification and operationalization of implementation strategies in real-world clinical settings is possible and provides examples and learnings for hospitals and health systems seeking to expand palliative care services by building a presence.

## Supplementary Information

Additional file 1: Topic guide (Key informants, periodic interviews). Semi-structured interview guide used during the early interviews with key informants with main questions of interest and prompts to get more in-depth information from study participants.

Additional file 2: Table S1. ERIC strategies and domains with definitions (combined and adapted from Powell et al., 2015 and Waltz et al., 2015). Definitions of the 73 ERIC strategies from Powell and colleagues (2015) combined with the table presented in Waltz and colleagues (2015) that organized the strategies into the nine identified clusters, along with mean importance and feasibility ratings, and go-zone quadrant. Additionally, we added a column for study-specific examples or definitions to help clarify and align understanding across the research team.

Additional file 3: Table S2. Summary of strategies used by sites A-F. Summary of how each site operationalized the strategies they employed, organized by domain. The number of strategies used within each domain is noted in parentheses after the domain name, and the number of strategies used by each site is shown in the last row of the table.

## Data Availability

The datasets generated and analyzed during the current study are not publicly available to maintain confidentiality for participating organizations, but are available from the corresponding author on reasonable request.

## Abbreviations

Cmty: Community
EMR: Electronic medical record
ERIC: Expert Recommendations for Implementing Change
Int: Interview
IP: Inpatient
OP: Outpatient
