# Supplementary material for: Building a presence: implementation strategies used to expand palliative care services across six diverse health systems in a longitudinal mixed methods study
Source: BMC Palliat Care. 2026 Apr 1;25:136. doi: 10.1186/s12904-026-02076-2 (PMC13169856; doi:10.1186/s12904-026-02076-2)
Supplement: Supplementary file 1 — Additional file 1: Topic guide (Key informants, periodic interviews). Semi-structured interview guide used during the early interviews with key informants with main questions of interest and prompts to get more in-depth information from study participants. [file 12904_2026_2076_MOESM1_ESM.docx]

Topic guide (Key informants, periodic interviews)

| **Main questions** | **Prompts** |
| --- | --- |
| **Progress so far** | |
| [Initial interview] Tell me about the program and how the program is going so far?  [Follow up interviews] How’s the program going? What’s happened with the program since we last spoke? | What changes to the program have been made and why?  Has your role changed?  In what ways are you trying to make [intervention] a routine part of practice?  How satisfied with the intervention/program are you? How satisfied do you think others are?  How well do you think this program/intervention fits with the existing standard of practice/workflows?  How has COVID shifted how things are moving or changing? |
| **Influences on implementation (barriers and facilitators)** | |
| Is there anything that has gone particularly well so far? | Why do you think it’s gone so well?  How will you try to build on that success? |
| Have you encountered any barriers during implementation? | Did you/how did you overcome them?  How did they change the program? |
| Is there anything you have learned from the other grantees? | Has the collaborative had any impact on your program? |
| **Perceptions of influence on outcomes and process** | |
| Do you think the program is having an impact on the Foundation’s goals (increasing goal concordant care, reducing suffering)? How/why not? | What kind of impact do you feel you’re having? Where are you having impact you wanted to, and where do you see it not having an impact and why?  How do you know it’s having an impact (or not)?  How could the impact be enhanced/ magnitude of impact increased? |
| What do you perceive as gaps in the care of seriously ill patients that still exist? |  |
| **Future expectations/ needs/ wishes** | |
| What are the next steps for the program? | What are your concerns going forward? |
