# Supplementary material for: Building a presence: implementation strategies used to expand palliative care services across six diverse health systems in a longitudinal mixed methods study
Source: BMC Palliat Care. 2026 Apr 1;25:136. doi: 10.1186/s12904-026-02076-2 (PMC13169856; doi:10.1186/s12904-026-02076-2)
Supplement: Supplementary file 2 — Additional file 2: Table S1. ERIC strategies and domains with definitions (combined and adapted from Powell et al., 2015 and Waltz et al., 2015). Definitions of the 73 ERIC strategies from Powell and colleagues (2015) combined with the table presented in Waltz and colleagues (2015) that organized the strategies into the nine identified clusters, along with mean importance and feasibility ratings, and go-zone quadrant. Additionally, we added a column for study-specific examples or definitions to help clarify and align understanding across the research team. [file 12904_2026_2076_MOESM2_ESM.docx]

Table S1. ERIC strategies and domains with definitions (combined and adapted from Powell et al., 2015 and Waltz et al., 2015)

| **Domain and Strategy Name** | **Definition** | **Importance** | **Feasibility** | **Go-zone quadrant** | **Study-specific examples or definitions** |
| --- | --- | --- | --- | --- | --- |
| **Use evaluative and iterative strategies (10 strategies)** |  | 4.19 | 4.01 | – |  |
| Assess for readiness and identify barriers and facilitators | Assess various aspects of an organization to determine its degree of readiness to implement, barriers that may impede implementation, and strengths that can be used in the implementation effort | 4.6 | 4.57 | I |  |
| Audit and provide feedback | Collect and summarize clinical performance data over a specified time period and give it to clinicians and administrators to monitor, evaluate, and modify provider behavior | 4.4 | 4.13 | I |  |
| Purposefully reexamine the implementation | Monitor progress and adjust clinical practices and implementation strategies to continuously improve the quality of care | 4.4 | 4.03 | I |  |
| Develop and implement tools for quality monitoring | Develop, test, and introduce into quality-monitoring systems the right input—the appropriate language, protocols, algorithms, standards, and measures (of processes, patient/consumer outcomes, and implementation outcomes) that are often specific to the innovation being implemented | 4.37 | 3.63 | I |  |
| Develop and organize quality monitoring systems | Develop and organize systems and procedures that monitor clinical processes and/or outcomes for the purpose of quality assurance and improvement | 4.33 | 3.37 | I |  |
| Develop a formal implementation blueprint | Develop a formal implementation blueprint that includes all goals and strategies. The blueprint should include the following: 1) aim/purpose of the implementation; 2) scope of the change (e.g., what organizational units are affected); 3) timeframe and milestones; and 4) appropriate performance/progress measures. Use and update this plan to guide the implementation effort over time | 4.3 | 4.47 | I |  |
| Conduct local needs assessment | Collect and analyze data related to the need for the innovation | 4.27 | 4.33 | I | Include assessment of needs and gaps to present to leadership |
| Stage implementation scale up | Phase implementation efforts by starting with small pilots or demonstration projects and gradually move to a system wide rollout | 3.97 | 3.77 | I |  |
| Obtain and use patients/ consumers and family feedback | Develop strategies to increase patient/consumer and family feedback on the implementation effort | 3.67 | 3.8 | I |  |
| Conduct cyclical small tests of change | Implement changes in a cyclical fashion using small tests of change before taking changes system-wide. Tests of change benefit from systematic measurement, and results of the tests of change are studied for insights on how to do better. This process continues serially over time, and refinement is added with each cycle | 3.63 | 4.03 | I |  |
| **Provide interactive assistance (4 strategies)** |  | 3.67 | 3.29 | – |  |
| Facilitation | A process of interactive problem solving and support that occurs in a context of a recognized need for improvement and a supportive interpersonal relationship | 4.13 | 3.77 | I |  |
| Provide local technical assistance | Develop and use a system to deliver technical assistance focused on implementation issues using local personnel | 3.97 | 3.2 | IV |  |
| Provide clinical supervision | Provide clinicians with ongoing supervision focusing on the innovation. Provide training for clinical supervisors who will supervise clinicians who provide the innovation | 3.83 | 3.1 | IV |  |
| Centralize technical assistance | Develop and use a centralized system to deliver technical assistance focused on implementation issues | 2.73 | 3.1 | III |  |
| **Adapt and tailor to context**  **(4 strategies)** |  | 3.59 | 3.3 | – |  |
| Tailor strategies | Tailor the implementation strategies to address barriers and leverage facilitators that were identified through earlier data collection | 4.37 | 4 | I |  |
| Promote adaptability | Identify the ways a clinical innovation can be tailored to meet local needs and clarify which elements of the innovation must be maintained to preserve fidelity | 3.9 | 3.57 | I | Well-defined components (e.g.., care navigation role) or interventions |
| Use data experts | Involve, hire, and/or consult experts to inform management on the use of data generated by implementation efforts | 3.23 | 3.13 | III |  |
| Use data warehousing techniques | Integrate clinical records across facilities and organizations to facilitate implementation across systems | 2.87 | 2.5 | III |  |
| **Develop stakeholder interrelationships**  **(17 strategies)** |  | 3.47 | 3.64 | – |  |
| Identify and prepare champions | Identify and prepare individuals who dedicate themselves to supporting, marketing, and driving through an implementation, overcoming indifference or resistance that the intervention may provoke in an organization | 4.2 | 3.77 | I |  |
| Organize clinician implementation team meetings | Develop and support teams of clinicians who are implementing the innovation and give them protected time to reflect on the implementation effort, share lessons learned, and support one another’s learning | 3.97 | 3.53 | I |  |
| Recruit, designate, and train for leadership | Recruit, designate, and train leaders for the change effort | 3.93 | 3.2 | IV |  |
| Inform local opinion leaders | Inform providers identified by colleagues as opinion leaders or “educationally influential” about the clinical innovation in the hopes that they will influence colleagues to adopt it | 3.9 | 4.03 | I |  |
| Build a coalition | Recruit and cultivate relationships with partners in the implementation effort | 3.77 | 3.63 | I |  |
| Obtain formal commitments | Obtain written commitments from key partners that state what they will do to implement the innovation | 3.77 | 3.17 | IV |  |
| Identify early adopters | Identify early adopters at the local site to learn from their experiences with the practice innovation | 3.7 | 3.7 | I |  |
| Conduct local consensus discussions | Include local providers and other stakeholders in discussions that address whether the chosen problem is important and whether the clinical innovation to address it is appropriate | 3.63 | 4.07 | I |  |
| Capture and share local knowledge | Capture local knowledge from implementation sites on how implementers and clinicians made something work in their setting and then share it with other sites | 3.63 | 3.87 | I |  |
| Use advisory boards and workgroups | Create and engage a formal group of multiple kinds of stakeholders to provide input and advice on implementation efforts and to elicit recommendations for improvements | 3.4 | 3.87 | I |  |
| Use an implementation advisor | Seek guidance from experts in implementation | 3.3 | 3.7 | I |  |
| Model and simulate change | Model or simulate the change that will be implemented prior to implementation | 3.3 | 3.2 | II |  |
| Visit other sites | Visit sites where a similar implementation effort has been considered successful | 3.17 | 3.73 | II |  |
| Involve executive boards | Involve existing governing structures (e.g., boards of directors, medical staff boards of governance) in the implementation effort, including the review of data on implementation processes | 2.97 | 3.63 | II |  |
| Develop an implementation glossary | Develop and distribute a list of terms describing the innovation, implementation, and stakeholders in the organizational change | 2.87 | 4.57 | II |  |
| Develop academic partnerships | Partner with a university or academic unit for the purposes of shared training and bringing research skills to an implementation project | 2.83 | 3.4 | II |  |
| Promote network weaving | Identify and build on existing high-quality working relationships and networks within and outside the organization, organizational units, teams, etc. to promote information sharing, collaborative problem-solving, and a shared vision/goal related to implementing the innovation | 2.7 | 2.77 | III |  |
| **Train and educate stakeholders (11 strategies)** |  | 3.43 | 3.93 | – |  |
| Conduct ongoing training | Plan for and conduct training in the clinical innovation in an ongoing way | 4.17 | 3.87 | I |  |
| Provide ongoing consultation | Provide ongoing consultation with one or more experts in the strategies used to support implementing the innovation | 4.17 | 3.63 | I |  |
| Develop educational materials | Develop and format manuals, toolkits, and other supporting materials in ways that make it easier for stakeholders to learn about the innovation and for clinicians to learn how to deliver the clinical innovation | 3.8 | 4.83 | I |  |
| Make training dynamic | Vary the information delivery methods to cater to different learning styles and work contexts, and shape the training in the innovation to be interactive | 3.67 | 4 | I |  |
| Distribute educational materials | Distribute educational materials (including guidelines, manuals, and toolkits) in person, by mail, and/or electronically | 3.5 | 4.77 | I |  |
| Use train-the-trainer strategies | Train designated clinicians or organizations to train others in the clinical innovation | 3.33 | 3.5 | I |  |
| Conduct educational meetings | Hold meetings targeted toward different stakeholder groups (e.g., providers, administrators, other organizational stakeholders, and community, patient/consumer, and family stakeholders) to teach them about the clinical innovation | 3.27 | 4.5 | I | Grand Rounds |
| Conduct educational outreach visits | Have a trained person meet with providers in their practice settings to educate providers about the clinical innovation with the intent of changing the provider's practice | 3.1 | 4.07 | II |  |
| Create a learning collaborative | Facilitate the formation of groups of providers or provider organizations and foster a collaborative learning environment to improve implementation of the clinical innovation | 3.1 | 3.43 | II |  |
| Shadow other experts | Provide ways for key individuals to directly observe experienced people engage with or use the targeted practice change/innovation | 2.87 | 3.37 | II |  |
| Work with educational institutions | Encourage educational institutions to train clinicians in the innovation | 2.73 | 3.3 | II |  |
|  |  |  |  |  |  |
| **Support clinicians**  **(5 strategies)** |  | 3.23 | 3.06 | – |  |
| Facilitate relay of clinical data to providers | Provide as close to real-time data as possible about key measures of process/outcomes using integrated modes/channels of communication in a way that promotes use of the targeted innovation | 4.17 | 3.43 | I |  |
| Remind clinicians | Develop reminder systems designed to help clinicians to recall information and/or prompt them to use the clinical innovation | 3.23 | 3.77 | II |  |
| Develop resource sharing agreements | Develop partnerships with organizations that have resources needed to implement the innovation | 3.07 | 3.13 | III |  |
| Revise professional roles | Shift and revise roles among professionals who provide care, and redesign job characteristics | 3 | 2.3 | III |  |
| Create new clinical teams | Change who serves on the clinical team, adding different disciplines and different skills to make it more likely that the clinical innovation is delivered (or is more successfully delivered) | 2.67 | 2.67 | III |  |
| **Engage consumers**  **(5 strategies)** |  | 3.25 | 2.95 | – |  |
| Involve patients/consumers and family members | Engage or include patients/consumers and families in the implementation effort | 3.87 | 3.63 | I |  |
| Intervene with patients/consumers to enhance uptake and adherence | Develop strategies with patients to encourage and problem solve around adherence | 3.5 | 3.07 | IV |  |
| Prepare patients/consumers to be active participants | Prepare patients/consumers to be active in their care, to ask questions, and specifically to inquire about care guidelines, the evidence behind clinical decisions, or about available evidence-supported treatments | 3.4 | 3.03 | IV | Prep patients to receive palliative care (patient acceptability) |
| Increase demand | Attempt to influence the market for the clinical innovation to increase competition intensity and to increase the maturity of the market for the clinical innovation | 3.3 | 2.33 | II |  |
| Use mass media | Use media to reach large numbers of people to spread the word about the clinical innovation | 2.17 | 2.7 | III |  |
| **Utilize financial strategies**  **(9 strategies)** |  | 2.86 | 2.09 | – |  |
| Fund and contract for the clinical innovation | Governments and other payers of services issue requests for proposals to deliver the innovation, use contracting processes to motivate providers to deliver the clinical innovation, and develop new funding formulas that make it more likely that providers will deliver the innovation | 3.67 | 2.43 | IV |  |
| Access new funding | Access new or existing money to facilitate the implementation | 3.57 | 2.4 | IV |  |
| Place innovation on fee for service lists/formularies | Work to place the clinical innovation on lists of actions for which providers can be reimbursed (e.g., a drug is placed on a formulary, a procedure is now reimbursable) | 3.4 | 2.1 | IV |  |
| Alter incentive/allowance structures | Work to incentivize the adoption and implementation of the clinical innovation | 3.17 | 2.23 | III |  |
| Make billing easier | Make it easier to bill for the clinical innovation | 2.93 | 1.77 | III |  |
| Alter patient/consumer fees | Create fee structures where patients/consumers pay less for preferred treatments (the clinical innovation) and more for less-preferred treatments | 2.6 | 2.03 | III |  |
| Use other payment schemes | Introduce payment approaches (in a catch-all category) | 2.3 | 1.87 | III |  |
| Develop disincentives | Provide financial disincentives for failure to implement or use the clinical innovations | 2.17 | 2.13 | III |  |
| Use capitated payments | Pay providers or care systems a set amount per patient/consumer for delivering clinical care | 1.97 | 1.8 | III |  |
| **Change infrastructure**  **(8 strategies)** |  | 2.4 | 2.01 | – |  |
| Mandate change | Have leadership declare the priority of the innovation and their determination to have it implemented | 3.23 | 2.63 | III |  |
| Change record systems | Change records systems to allow better assessment of implementation or clinical outcomes | 2.83 | 2.23 | III | algorithm to generate referrals |
| Change physical structure and equipment | Evaluate current configurations and adapt, as needed, the physical structure and/or equipment (e.g., changing the layout of a room, adding equipment) to best accommodate the targeted innovation | 2.6 | 2.27 | III |  |
| Create or change credentialing and/or licensure standards | Create an organization that certifies clinicians in the innovation or encourage an existing organization to do so. Change governmental professional certification or licensure requirements to include delivering the innovation. Work to alter continuing education requirements to shape professional practice toward the innovation | 2.23 | 1.47 | III |  |
| Change service sites | Change the location of clinical service sites to increase access | 2.2 | 2.2 | III |  |
| Change accreditation or membership requirements | Strive to alter accreditation standards so that they require or encourage use of the clinical innovation. Work to alter membership organization requirements so that those who want to affiliate with the organization are encouraged or required to use the clinical innovation | 2.17 | 1.8 | III |  |
| Start a dissemination organization | Identify or start a separate organization that is responsible for disseminating the clinical innovation. It could be a for-profit or non-profit organization | 2.03 | 2.13 | III |  |
| Change liability laws | Participate in liability reform efforts that make clinicians more willing to deliver the clinical innovation | 1.87 | 1.33 | III |  |
