# Supplementary material for: Building a presence: implementation strategies used to expand palliative care services across six diverse health systems in a longitudinal mixed methods study
Source: BMC Palliat Care. 2026 Apr 1;25:136. doi: 10.1186/s12904-026-02076-2 (PMC13169856; doi:10.1186/s12904-026-02076-2)
Supplement: Supplementary file 3 — Additional file 3: Table S2. Summary of strategies used by sites A-F. Summary of how each site operationalized the strategies they employed, organized by domain. The number of strategies used within each domain is noted in parentheses after the domain name, and the number of strategies used by each site is shown in the last row of the table. [file 12904_2026_2076_MOESM3_ESM.docx]

Table S2. Summary of strategies used by sites A-F

|  | **A: outpatient (OP)** | **B: outpatient** | **C: inpatient** | **D: community** | **E: cmty pilots** | **F: OP pilot** |
| --- | --- | --- | --- | --- | --- | --- |
| **Utilize financial strategies** (1 of 9 strategies used) | | | | | | |
| *Access new funding* | Answered request for proposals for 3-year expansion, spread, or pilot palliative care services from the Foundation, and granted full or partial funding | | | | | |
| **Use evaluative and iterative strategies** (7 of 10 strategies used) | | | | | | |
| *Develop a formal implementation blueprint* | Outlined services and eligibility, predicted reach, project plan, leadership and staffing | Described project plan with quarterly implementation milestones, predicted reach, leadership and staffing | Detailed current services, 6-year vision, 3-year implementation plan with quarterly milestones, leadership and staffing, and predicted reach | Detailed current services, 6-year vision, 3-year implementation plan with quarterly activities and milestones, leadership and staffing, and predicted reach | Presented current services, 6-year vision, 3-year implementation plan with quarterly activities, leadership and staffing, and desired outcomes | Described current services, predicted reach, 6-year vision, 3-year plan and implementation milestones, leadership and staffing |
| *Assess for readiness and identify barriers and facilitators* | Included facilitators and potential risks or barriers in proposal | Outlined facilitators and potential risks, and how to minimize risks | Included risk assessment & impact of risks in proposal | Outlined potential risks & mitigation strategies, and facilitators in proposal | Included risks, facilitators, and mitigation strategy in proposal | Included a risk assessment in proposal |
| *Conduct local needs assessment* |  |  |  |  |  | Surveyed primary care physicians about clinician and patient needs |
| *Audit and provide feedback* |  |  |  | Tracked provider referral patterns and shared data with offices  Tracked and shared number of consults with team |  |  |
| *Conduct cyclical small tests of change* |  |  |  |  | Piloted two different home-based service teams to serve hospice discharges and non-admits |  |
| *Stage implementation scale-up* |  |  |  |  |  | Piloted tiered system in primary care, then moving to oncology |
| *Purposefully reexamine the implementation* |  |  |  |  |  | Stepped-wedge design with three cohorts, discussing lessons learned and changes between cohorts |
| **Support clinicians** (3 of 5 strategies used) | | | | | | |
| *Create new clinical teams* | Hired NP; unable to hire palliative care physician | Hired physician director and NP, with existing social worker and care coordinator.  All positions were vacated spring/ summer 2021; new physician director found with inpatient NP support | Onboarded palliative care nurse champions  Hired palliative care physician, NPs, RNs, and chaplain. Physician oversees both inpatient services, Site 1 & 2; each site staffed with a palliative care NP, RN, and chaplain | Hired palliative care physician, NP, social worker and care coordinator, with existing clinic manager | Pilot 1: hired NP, patient coordinator, and social worker; onboarded pharmacist, physician, and manager for home-based pilot  Pilot 2: hired nurse and social worker, with existing physician to oversee home-based pilot  Pilot 3: onboarded existing community navigator  Pilot 4: hired social worker for caregiver support | Hired patient navigator, with support from existing outpatient palliative care physicians, social worker, and chaplain |
| *Revise professional roles* | During COVID, NP work shifted to focus on testing and vaccinations  Without physician hire, NP led efforts with inpatient NP mentorship  In the absence of more patients, NP integrated into cancer center as care coordinator |  | Implementation team assisted with COVID surge planning and telehealth efforts  Palliative care nurse champion role shifted to dedicated palliative care nurse (RN) | Reorganization and restructuring occurred; clinic manager was acting project manager, and their role was split between 3 others | Pilot 2: With additional after-hours program, palliative care physicians and nurses were on rotating coverage | Patient navigator role modified to provide a certain level of emotional support before referral to social worker rather than immediate referral |
| *Remind clinicians* |  |  | Flyers with palliative care’s contact information | Algorithm-generated patient lists sent to referring providers |  |  |
| **Develop stakeholder relationships** (6 of 17 strategies used) | | | | | | |
| *Involve executive members* | Executive sponsors named in proposal, tasked with financial and operational support | Hospital leadership and administration, fully supported proposal, with named executive sponsors  Invited and engaged new CNO/CNS to participate in implementation discussions and interviews | Executive sponsors named in proposal  Strong active sponsorship from start to finish: involved in program implementation discussions and activities  Executives invited to implementation committee advisory group  One-on-one conversations with new executives about the program | Regional and hospital campus executives pledged support  Ongoing conversations with leadership to share work and advocate for the continued spread of palliative care services | Regional and local leaders involved during grant planning and contributed in-kind funding  Annual or biannual meetings with regional and local leaders to share progress and learnings | Executive sponsors named in proposal |
| *Organize clinician implementation team meetings* |  | Meetings on as needed basis; modified as team personnel changed | Weekly team meetings to discuss implementation efforts. Team included project coordinator, physicians, social worker, and NPs | Weekly team meetings with physician, NP, and clinic manager, looping in consultant as needed | Overseeing implementation team met with pilot leads periodically to discuss updates and progress | Weekly/biweekly team meetings, with physicians, care navigator, and project manager |
| *Identify and prepare champions* |  |  | Nurse champions identified and trained to provide palliative care service initially  ICU nurse champion had daily check-ins with team and helped build awareness within the unit | Clinic nurses, social workers, and/or staff identified as champions, to encourage and prompt physician referral |  | Physician champions at primary practice were engaged early on and through, helping assess needs and getting buy-in among colleagues for the program |
| *Use advisory boards* |  |  | Organized an implementation committee to include C-suite members, and quality and nursing directors to provide updates, share learnings, and get input |  |  | Scheduled existing internal experts to provide input on initial plans, materials, and personnel responsibilities |
| *Promote network weaving* |  | Reached out to existing internal department relationships (i.e., cancer, ED, and cardiology) to create better referral processes |  |  |  |  |
| *Develop academic partnerships* | In the absence of hiring physician lead, pivoted and contracted with academic unit for training and consultation. |  |  |  |  |  |
| **Train and educate stakeholders** (8 of 11 strategies used) | | | | | | |
| *Conduct educational meetings* |  | Tumor boards  Department meetings, including cardiology and oncology | Grand Rounds  Department meetings, including the ICU | Department meetings, with primary care, oncology, cardiology, pulmonology, neurology, and nephrology | Meetings with physicians about the pilots, and who to refer | Clinic meetings with physicians about the pilot |
| *Conduct educational outreach visits* | NP shadowed oncologist to better understand workflow and referral process |  | Nurse huddles and rounds with clinicians | Oncology clinic visits with nurses and social workers |  |  |
| *Develop educational materials* |  | Brochure on palliative care service and how they can provide that extra layer of support | Instruction sheet on where to document advance care planning conversations |  | Four module educational program on communication skills and symptom management issues |  |
| *Distribute educational materials* |  | Laid out brochures for patients and providers | Posted instruction sheet in provider workrooms |  | Provided materials and training to providers |  |
| *Conduct ongoing trainings* | Training and mentorship for new outpatient NP from inpatient NP |  | Trainings for physicians about how to consult or refer to service  Trainings for nurses to become palliative care champions or advocates |  |  | Continuous training and mentorship for patient navigator by palliative care team members |
| *Use train-the-trainer strategies* |  |  | Train palliative care nurses on palliative communication strategies to then train other nurses | Primary palliative care trainings for non-palliative care clinicians done by trained physicians |  |  |
| *Make training dynamic* |  |  | Hospital-wide trainings, in-house mentorship with case-by-case patient training |  |  |  |
| *Create a learning collaborative* | Funding organization created a learning collaborative early on. In those nine sessions, sites shared initial learnings and challenges. It helped connect a community hospital with the academic center for support, and there was some outreach between sites when there were similar ideas or interest in what other, more established programs were doing, further facilitated by the evaluators/ authors of this paper. | | | | | |
| **Adapt and tailor to context** (2 of 4 strategies used) | | | | | | |
| *Promote adaptability* |  |  |  | Changed name of the program and redesigned materials based on physician feedback and new guidelines |  | Team piloted and adapted the care navigator script |
| *Use data warehousing techniques* |  |  | Transitioned to new medical record system to be used across the health system |  | Pilot 4: Affiliate electronic medical record access for community navigators to communicate with physicians |  |
| **Change infrastructure** (2 of 8 strategies used) | | | | | | |
| *Change record systems* |  |  |  | Developed eligibility tool to identify palliative care eligible patients, though found many were not the best fit upon physician review | Built algorithms to identify: 1) patients with caregivers that could benefit from additional support, used to supplement referrals; and 2) patients with high social needs | Created an algorithm as a screening tool to identify eligible patients for the tiered-model of outpatient palliative care, which underwent 3 rounds of clinical validation |
| *Create or change credentialing and/or licensure standards* |  |  | Secured continuing education (CE) credits for attendees of nurse champion trainings |  |  |  |
| **Engage consumers** (2 of 5 strategies used) | | | | | | |
| *Prepare patients to be active participants* | NP educated patients about palliative care services, allowing them to decide whether to proceed or not |  |  |  |  | Patient navigator provided advance care planning information and resources to patients |
| *Use mass media* | Palliative care education through community radio station | Advertised palliative care service in local newspaper and television and YouTube channel |  |  |  |  |
| **Provide interactive assistance** (2 of 4 strategies used) | | | | | | |
| *Facilitation* |  |  | Care coordinator liaised, and participated in problem solving with service team | Physician consultant provided guidance and support to the implementation team |  |  |
| *Centralize technical assistance* |  |  | Implementation team kept programs accountable and helped troubleshoot any issues for the inpatient services |  |  |  |
| **Number of implementation strategies used:** | **11** | **12** | **23** | **17** | **14** | **17** |

***cational meetings*** a***trainings*** with referring clinicians were planned for Powell, B. J., 1007/s11414-015-9475-6 Beidas, R. S., Lewis, C. C., Aarons, G. A., McMillen, J. C., Proctor, E. K., & Mandell, D. S. (2017). Methods to improve the selection and tailoring of implementation strategies. The journal of behavioral health services & research, 44, 177-194. everyprogram, but werhindered by the COVID-19 pandemiEducatio
